# Risk factors for treatment resistance among women with postpartum depression in a nationwide study

**DOI:** 10.1038/s44220-026-00587-8

**Published:** 2026-02-04

**Authors:** Yufeng Chen, Emma Bränn, Marie Bendix, Emily E. Joyce, Emma Fransson, Yi Lu, Alkistis Skalkidou, Donghao Lu

**Affiliations:** 1 Unit of Integrative Epidemiology, Institute of Environmental Medicine, Karolinska Institutet, Stockholm, Sweden; 2 Centre for Epidemiology and Community Medicine, Region Stockholm, Stockholm, Sweden; 3 Department of Clinical Neuroscience, Centre for Psychiatry Research, Karolinska Institutet, Stockholm Health Care Services, Stockholm, Sweden; 4 Department of Clinical Sciences/Psychiatry, Umeå University, Umeå, Sweden; 5 Department of Women’s and Children’s Health, Uppsala University, Uppsala, Sweden; 6 Department of Microbiology, Tumor and Cell Biology, Karolinska Institutet, Stockholm, Sweden; 7 Department of Medical Epidemiology and Biostatistics, Karolinska Institutet, Stockholm, Sweden

**Keywords:** Epidemiology, Depression, Risk factors

## Abstract

The occurrence of treatment resistance in women with postpartum depression (PPD) and risk factors for treatment resistance remain less studied. This study aimed to determine the rate of treatment resistance and the associated risk factors among women with PPD in a nationwide setting. Here we conducted a nationwide register-based cohort study of 58,618 patients with a first-ever PPD during 2006–2021 in Sweden. Information on demographics, pregnancy characteristics, pre-existing physical and psychiatric conditions and treatment was retrieved from Swedish national registers. The outcome was treatment-resistant PPD (TRPPD) within 1 year following PPD diagnosis. Associations between potential risk factors and TRPPD were assessed using multivariable Poisson regression. Among the 58,618 patients with PPD, 3,522 (6.0%) met the criteria for TRPPD during 1 year after PPD diagnosis. Lower educational level, lower household income, being non-cohabiting, smoking in early pregnancy, delivery by cesarean section, pre-existing physical conditions and pre-existing psychiatric disorders were significantly associated with a higher risk of TRPPD. In addition, patients with two births (versus primiparity) or with a prior premenstrual disorder had a lower risk of TRPPD. Treatment resistance in patients with PPD is common and is notably associated with specific demographic and clinical profiles. These findings may provide grounds for practical risk assessment at PPD diagnosis and highlight the need for personalized management strategies.

## Abstract (web summary)

Chen et al. assessed the prevalence and risk factors for treatment-resistant postpartum depression using nationwide data from the Swedish National Medical Birth Register.

## Main

Postpartum depression (PPD) impacts millions of new mothers worldwide, with approximately one out of five mothers experiencing a depressive episode within the first months after delivery^1^. PPD has substantial influence on the affected patients in both the short and the long run^2^, resulting in negative consequences (for example, increased suicidal behavior^3^ and premature death^4^) and increased societal burden^5^. Psychotherapy is the first-line treatment for mild PPD, while pharmacotherapy is often indicated for patients with moderate and severe symptoms^6^. Although selective serotonin reuptake inhibitors are the most prescribed and first choice medications for PPD^6,7^, evidence from randomized clinical trials^8^ shows that the response rates are 43%–87% and remission rates are 37%–65% after 6–8 weeks of treatment.

Most existing studies define treatment resistance as no symptom remission after two or more pharmacologically different antidepressant treatments at an adequate dose and duration^9^. Clinical studies on non-perinatal depression report that a substantial number of patients^10^ (~30%) develop treatment resistance, while epidemiological studies indicate 11%–15% of patients are classified with treatment resistance^7,11,12^. Treatment-resistant depression has been linked to negative personal and societal outcomes^11–13^ and excess mortality^12,14^. Varying factors, including comorbid psychiatric conditions, sociodemographic vulnerability (for example, younger age, unemployment), medical conditions and greater severity of depression, have been associated with treatment-resistant depression^15^.

By contrast, limited data exist regarding how often treatment resistance occurs in patients with PPD, a subset of depression that may involve distinct pathophysiological mechanisms^16^, such as reproductive hormonal fluctuations^17^. In addition, PPD is often undertreated^6,18^ due to concerns about the potential adverse effects of pharmacotherapy on the infant, potentially leading to different clinical profiles and courses compared with non-perinatal depression. In addition, the clinical prognosis of PPD might be attributable to pregnancy characteristics and outcomes^19–21^, which are not relevant in the context of non-perinatal depression. The exclusive report, to our knowledge, a descriptive study in the United States with 3.2 million pregnant women, reported that about 5% of the patients with perinatal depression developed treatment resistance within 1 year^22^. However, the rates of PPD (2.5%) and treatment resistance in this study may have been underestimated as the study population consisted of only patients with commercial insurance, a population with better socioeconomic status^22^. Given the high disease burden of PPD^3,4^ and the occurrence of treatment resistance, which potentially leads to worse prognosis, it is critical to identify risk factors that influence the treatment response. However, to our knowledge, risk factors for treatment resistance have not been well investigated.

To address these knowledge gaps, we leveraged a national register-based cohort of women diagnosed with PPD in Sweden to assess the prevalence of treatment resistance and to identify potential risk factors associated with treatment resistance.

## Results

**Fig. 1:**
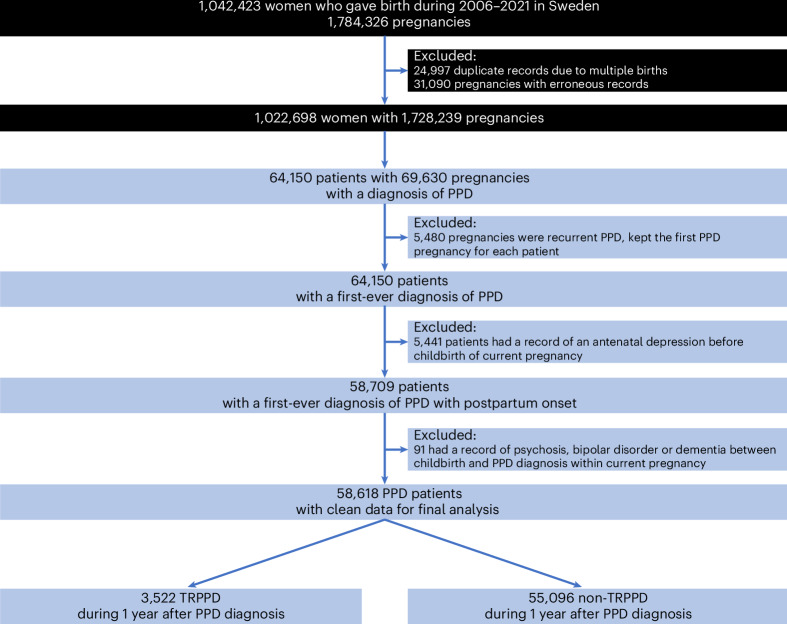
Study design. Pregnant women were identified from the Swedish Medical Birth Register. Depression was ascertained from the National Patient Register (Inpatient/Outpatient specialized care), Prescribed Drug Register and five regional primary care centers.

We identified 64,150 patients with a diagnosis of first-ever PPD. After excluding 5,441 patients with a record of antepartum depression and 91 with psychosis, bipolar disorder or dementia between childbirth and PPD diagnosis, 58,618 patients remained for the final analysis (Fig. 1). Among them (mean age 30.8 years, s.d. 5.3 years), 50,679 (86.5%) had received treatments (antidepressants or add-on medications or electroconvulsive therapy (ECT) or repetitive transcranial magnetic stimulation (rTMS)), and 3,522 (6.0%) fulfilled the criteria of TRPPD within 1 year after PPD diagnosis.

### Demographic and lifestyle factors

Compared with patients without TRPPD, patients with TRPPD were more likely to be born outside Sweden (adjusted risk ratio (RR) for Europe versus Sweden: 1.25, 95% confidence interval (CI) 1.09–1.42; non-Europe versus Sweden: 1.15, 95% CI 1.04–1.28) and reside in the north of the country (northern versus southern: 1.17, 1.05–1.29), and were less likely to be diagnosed with PPD after 2015 (0.85, 0.78–0.93; Table 1). Patients with lower educational attainment (<9 versus >12 y: 1.61, 1.45–1.79) or lower household income level (lowest 20% versus top 20%: 1.35, 1.20–1.52) and being non-cohabiting (1.27, 1.15–1.42) had higher risks of TRPPD. The risk of TRPPD was also higher in patients who were smoking in early pregnancy (RRs for 1–9 cigarettes day^−1^: 1.45, 1.30–1.63; ≥10 cigarettes day^−1^: 1.53, 1.28–1.84) than in non-smoking patients (Table 1).

**Table 1:** Demographic and lifestyle characteristics associated with treatment resistance among patients with PPD

Variables	Non-TRPPD	TRPPD		RR (95% CI)^b^			
	(*n* = 55,096)	(*n* = 3,522)	AR (%)^a^	Model 1	*P*^c^	Model 2	*P*^c^
**Maternal age, y**							
<20	849 (1.5)	85 (2.4)	9.1	1.52 (1.22–1.90)	<0.001*	1.07 (0.85–1.36)	0.743
20–24	6,962 (12.6)	575 (16.3)	7.6	1.27 (1.15–1.41)	<0.001*	1.10 (0.99–1.22)	0.145
25–29	16,391 (29.7)	1,044 (29.6)	6.0	reference		reference	
30–34	18,577 (33.7)	1,061 (30.1)	5.4	0.90 (0.83–0.98)	0.032*	0.96 (0.88–1.04)	0.494
35–39	9,925 (18.0)	605 (17.2)	5.7	0.96 (0.87–1.06)	0.546	1.01 (0.91–1.12)	0.931
≥40	2,392 (4.3)	152 (4.3)	6.0	1.00 (0.84–1.18)	0.984	1.01 (0.85–1.20)	0.941
**Educational level, y**							
>12	25,298 (45.9)	1,296 (36.8)	4.9	reference		reference	
9–12	21,788 (39.5)	1,454 (41.3)	6.3	1.28 (1.19–1.38)	<0.001*	1.24 (1.14–1.34)	<0.001*
<9	6,921 (12.6)	651 (18.5)	8.6	1.76 (1.61–1.94)	<0.001*	1.61 (1.45–1.79)	<0.001*
Unknown	1,089 (2.0)	121 (3.4)	10.0	NA		NA	
**Household income level**							
Top 20%	10,068 (18.3)	489 (13.9)	4.6	reference		reference	
Middle	34,318 (62.3)	2,159 (61.3)	5.9	1.28 (1.16–1.41)	<0.001*	1.19 (1.07–1.31)	0.002*
Lowest 20%	10,426 (18.9)	842 (23.9)	7.5	1.61 (1.44–1.80)	<0.001*	1.35 (1.20–1.52)	<0.001*
Unknown	284 (0.5)	32 (0.9)	10.1	NA		NA	
**Civil status**							
Cohabiting	50,636 (91.9)	3,103 (88.1)	5.8	reference		reference	
Non-cohabiting	4,460 (8.1)	419 (11.9)	8.6	1.49 (1.34–1.65)	<0.001*	1.27 (1.15–1.42)	<0.001*
**Calendar year at delivery**							
2006–2010	14,341 (26.0)	1,015 (28.8)	6.6	reference		reference	
2011–2015	17,074 (31.0)	1,153 (32.7)	6.3	0.95 (0.87–1.03)	0.220	0.97 (0.89–1.06)	0.536
2016–2021	23,681 (43.0)	1,354 (38.4)	5.4	0.81 (0.74–0.88)	<0.001*	0.85 (0.78–0.93)	<0.001*
**Maternal country of birth**							
Sweden	45,795 (83.1)	2,809 (79.8)	5.8	reference		reference	
Europe	3,237 (5.9)	254 (7.2)	7.3	1.26 (1.11–1.43)	0.001*	1.25 (1.09–1.42)	0.003*
Non-Europe	6,064 (11.0)	459 (13.0)	7.0	1.22 (1.10–1.34)	<0.001*	1.15 (1.04–1.28)	0.015*
**Maternal residency in Sweden**							
Southern	12,068 (21.9)	762 (21.6)	5.9	reference		reference	
Middle	33,948 (61.6)	2,082 (59.1)	5.8	0.97 (0.90–1.06)	0.621	1.00 (0.92–1.08)	0.955
Northern	9,080 (16.5)	678 (19.3)	6.9	1.17 (1.05–1.30)	0.006*	1.17 (1.05–1.29)	0.008*
**Body mass index in early pregnancy, kg** **m**^**−2**^							
<18.5	1,254 (2.3)	98 (2.8)	7.2	1.25 (1.02–1.54)	0.050	1.16 (0.95–1.42)	0.288
18.5–<25	27,918 (50.7)	1,713 (48.6)	5.8	reference		reference	
25–<30	13,719 (24.9)	869 (24.7)	6.0	1.03 (0.95–1.12)	0.592	1.01 (0.93–1.09)	0.941
30–<40	7,676 (13.9)	539 (15.3)	6.6	1.13 (1.03–1.25)	0.019*	1.07 (0.97–1.18)	0.300
≥40	845 (1.5)	60 (1.7)	6.6	1.15 (0.89–1.48)	0.424	1.05 (0.81–1.36)	0.833
Unknown	3,684 (6.7)	243 (6.9)	6.2	NA		NA	
**Smoking in early pregnancy**							
No smoking	47,360 (86.0)	2,799 (79.5)	5.6	reference		reference	
1–9 cigarettes day^−1^	3,592 (6.5)	379 (10.8)	9.5	1.71 (1.54–1.90)	<0.001*	1.45 (1.30–1.63)	<0.001*
≥10 cigarettes day^−1^	1,168 (2.1)	132 (3.7)	10.2	1.82 (1.53–2.17)	<0.001*	1.53 (1.28–1.84)	<0.001*
Unknown	2,976 (5.4)	212 (6.0)	6.6	NA		NA	
**Snus use in early pregnancy**							
No	51,919 (94.2)	3,293 (93.5)	6.0	reference		reference	
Yes	12,01 (2.2)	92 (2.6)	7.1	0.84 (0.68–1.03)	0.154	0.91 (0.74–1.12)	0.562
Unknown	1,976 (3.6)	137 (3.9)	6.5	NA		NA	

NA, not applicable.

^a^Absolute risk of TRPPD = (number of TRPPD/total number of patients with PPD in this category) × 100%.

^b^Model 1: estimates from Poisson regression, univariate analysis without adjustment; Model 2: estimates from Poisson regression, adjusted for age, educational level, calendar year, residential region, maternal country of birth, parity and multiple gestation, if applicable.

^c^*P* values were corrected for multiple testing using Benjamini-Hochberg method.

*P < 0.05.

### Pregnancy characteristics

We observed a lower risk of TRPPD in patients having two births (RR 0.89, 0.82–0.96) but not in patients having three or more births, compared with primiparous patients (Table 2). Patients with cesarean section (RR 1.15, 1.07–1.25) and patients with a preterm delivery (32–36 weeks; RR 1.23, 1.07–1.40) were at a higher risk of TRPPD. No associations were noted for other pregnancy outcomes, including multiple gestation, birth weight, Apgar score at 5 min and stillbirth (Table 2).

**Table 2:** Pregnancy-related characteristics associated with treatment resistance among patients with PPD

Variables	Non-TRPPD	TRPPD		RR (95% CI)^b^			
	(*n* = 55,096)	(*n* = 3,522)	AR (%) ^a^	Model 1	*P*^c^	Model 2	*P*^c^
**Parity**							
1	25,619 (46.5)	1,696 (48.2)	6.2	reference		reference	
2	19,843 (36.0)	1,141 (32.4)	5.4	0.88 (0.81–0.94)	0.001*	0.89 (0.82–0.96)	0.006*
≥3	9,634 (17.5)	685 (19.4)	6.6	1.07 (0.98–1.17)	0.215	1.01 (0.91–1.11)	0.941
**Multiple gestation**							
No	54,057 (98.1)	3,456 (98.1)	6.0	reference		reference	
Yes	1,039 (1.9)	66 (1.9)	6.0	0.99 (0.78–1.27)	0.984	1.01 (0.79–1.29)	0.943
**Delivery mode**							
Non-assisted vaginal	39,171 (71.1)	2,425 (68.9)	5.8	reference		reference	
Assisted vaginal	3,516 (6.4)	218 (6.2)	5.8	1.00 (0.87–1.15)	0.984	1.01 (0.88–1.17)	0.931
Cesarean section	12,409 (22.5)	879 (25)	6.6	1.13 (1.05–1.23)	0.003*	1.15 (1.07–1.25)	0.001*
**Gestational length, weeks**							
22–31	882 (1.6)	53 (1.5)	5.7	0.96 (0.73–1.25)	0.797	0.92 (0.70–1.21)	0.743
32–36	2,998 (5.4)	242 (6.9)	7.5	1.26 (1.10–1.44)	0.001*	1.23 (1.07–1.40)	0.006*
37–41	48,123 (87.3)	3,034 (86.1)	5.9	reference		reference	
42–46	3,083 (5.6)	192 (5.5)	5.9	0.99 (0.85–1.14)	0.923	0.99 (0.86–1.15)	0.943
Unknown	10 ( < 0.1)	<5 (<0.1)	9.1	NA		NA	
**Birth weight, g**							
<1,500	768 (1.4)	41 (1.2)	5.1	0.85 (0.62–1.15)	0.423	0.81 (0.60–1.11)	0.321
1,500–<2,500	2,150 (3.9)	157 (4.5)	6.8	1.14 (0.97–1.33)	0.184	1.09 (0.92–1.28)	0.494
≥2,500	52,076 (94.5)	3,316 (94.2)	6.0	reference		reference	
Unknown	102 (0.2)	8 (0.2)	7.3	NA		NA	
**Apgar score at 5** **min**							
≥7	53,152 (96.5)	3,393 (96.3)	6.0	reference		reference	
<7	1,525 (2.8)	105 (3.0)	6.4	1.07 (0.88–1.30)	0.592	1.04 (0.86–1.26)	0.833
Unknown	419 (0.8)	24 (0.7)	5.4	NA		NA	
**Stillbirth**							
No	54,544 (99.0)	3,490 (99.1)	6.0	reference		reference	
Yes	552 (1.0)	32 (0.9)	5.5	0.91 (0.64–1.29)	0.680	0.86 (0.61–1.22)	0.593

^a^Absolute risk of TRPPD = (number of TRPPD/total number of patients with PPD in this category) × 100%.

^b^Model 1: estimates from Poisson regression, univariate analysis without adjustment.

Model 2: estimates from Poisson regression, adjusted for age, educational level, calendar year, residential region, maternal country of birth, parity and multiple gestation, if applicable.

^c^*P* values were corrected for multiple testing using Benjamini-Hochberg method.

*P < 0.05.

### Physical and psychiatric conditions existing before PPD

Pre-existing physical conditions (RRs for Charlson comorbidity index = 1: 1.28, 1.16–1.42; Charlson comorbidity index ≥ 2: 1.42, 1.17–1.73) were associated with higher risks of TRPPD, but no association was found with hypertensive disorders or diabetes when assessed separately (Table 3). In addition, patients with any pre-existing psychiatric disorder were at a higher risk of TRPPD, with RR of 2.21 (2.06–2.37). The RRs for specific types of psychiatric disorders ranged from 1.69 (1.53–1.87) for substance abuse to 8.49 (7.58–9.52) for psychotic disorders (Table 3). Patients with a prior premenstrual disorder had a lower risk of TRPPD (0.71, 0.59–0.86).

**Table 3:** Pre-existing medical conditions associated with treatment resistance among patients with PPD

Variables	Non-TRPPD	TRPPD		RR (95% CI)^b^			
	(*n* = 55,096)	(*n* = 3,522)	AR (%) ^a^	Model 1	*P*^c^	Model 2	*P*^c^
**Hypertension**							
No	52,603 (95.5)	3,343 (94.9)	6.0	reference		reference	
Essential hypotension	434 (0.8)	31 (0.9)	6.7	1.12 (0.78–1.59)	0.640	1.12 (0.79–1.60)	0.721
Pre-eclampsia	2,059 (3.7)	148 (4.2)	6.7	1.12 (0.95–1.32)	0.255	1.12 (0.95–1.32)	0.321
**Diabetes**							
No	53,469 (97.0)	3,421 (97.1)	6.0	reference		reference	
Gestational diabetes	1,131 (2.1)	80 (2.3)	6.6	1.10 (0.88–1.37)	0.541	1.09 (0.87–1.36)	0.636
Pregestational diabetes	496 (0.9)	21 (0.6)	4.1	0.68 (0.44–1.04)	0.122	0.66 (0.43–1.01)	0.109
**Charlson comorbidity index**							
0	48,601 (88.2)	2,985 (84.8)	5.8	reference		reference	
1	5,320 (9.7)	431 (12.2)	7.5	1.30 (1.17–1.43)	<0.001*	1.28 (1.16–1.42)	<0.001*
≥2	1,175 (2.1)	106 (3.0)	8.3	1.43 (1.18–1.74)	0.001*	1.42 (1.17–1.73)	0.001*
**Psychiatric disorder, any**							
No	35,057 (63.6)	1,508 (42.8)	4.1	reference		reference	
Yes	20,039 (36.4)	2,014 (57.2)	9.1	2.21 (2.07–2.37)	<0.001*	2.21 (2.06–2.37)	<0.001*
**Psychotic disorders**							
No	54,755 (99.4)	3,186 (90.5)	5.5	reference		reference	
Yes	341 (0.6)	336 (9.5)	49.6	9.03 (8.07–10.1)	<0.001*	8.49 (7.58–9.52)	<0.001*
**Sleep disorders**							
No	54,426 (98.8)	3419 (97.1)	5.9	reference		reference	
Yes	670 (1.2)	103 (2.9)	13.3	2.25 (1.85–2.74)	<0.001*	2.11 (1.73–2.57)	<0.001*
**Personality disorder**							
No	53,480 (97.1)	3,280 (93.1)	5.8	reference		reference	
Yes	1,616 (2.9)	242 (6.9)	13.0	2.25 (1.98-2.57)	<0.001*	2.12 (1.85–2.42)	<0.001*
**Developmental and/or neuropsychiatric disorders**							
No	52,299 (94.9)	3,178 (90.2)	5.7	reference		reference	
Yes	2,797 (5.1)	344 (9.8)	11.0	1.91 (1.71–2.14)	<0.001*	1.77 (1.57–1.99)	<0.001*
**Depressive disorder**							
No	47,695 (86.6)	2,748 (78.0)	5.4	reference		reference	
Yes	7,401 (13.4)	774 (22.0)	9.5	1.74 (1.60–1.88)	<0.001*	1.72 (1.58–1.86)	<0.001*
**Anxiety**							
No	45,317 (82.3)	2,516 (71.4)	5.3	reference		reference	
Yes	9,779 (17.7)	1,006 (28.6)	9.3	1.77 (1.65–1.91)	<0.001*	1.76 (1.63–1.90)	<0.001*
**Substance abuse**							
No	51,135 (92.8)	3,067 (87.1)	5.7	reference		reference	
Yes	3,961 (7.2)	455 (12.9)	10.3	1.82 (1.65–2.01)	<0.001*	1.69 (1.53–1.87)	<0.001*
**Stress-related disorders**							
No	49,539 (89.9)	2,891 (82.1)	5.5	reference		reference	
Yes	5,557 (10.1)	631 (17.9)	10.2	1.85 (1.70–2.02)	<0.001*	1.77 (1.62–1.93)	<0.001*
**Premenstrual disorder**							
No	52,279 (94.9)	3,409 (96.8)	6.1	reference		reference	
Yes	2,817 (5.1)	113 (3.2)	3.9	0.63 (0.52–0.76)	<0.001*	0.71 (0.59–0.86)	0.001*

^a^Absolute risk of TRPPD = (number of TRPPD/total number of patients with PPD in this category) × 100%.

^b^Model 1: estimates from Poisson regression, univariate analysis without adjustment. Model 2: estimates from Poisson regression, adjusted for age, educational level, calendar year, residential region, maternal country of birth, parity and multiple gestation, if applicable.

^c^*P* values were corrected for multiple testing using Benjamini-Hochberg method.

*P < 0.05.

### Sensitivity analyses

We observed comparable associations when restricted to PPD patients without any pre-existing psychiatric disorders, except that the association with having two births was no longer present, while a higher risk of TRPPD was noted among patients with three or more births (Supplementary Table 1). Similar results were obtained when restricting the analysis to PPD ascertained through clinical diagnosis only or restricting to counties with primary care data available (Supplementary Table 2). Using the two alternative definitions of TRPPD also yielded comparable results (Supplementary Table 2). Last, in the mutually adjusted model, we found all primary factors remained statistically significant despite the associations being somewhat attenuated (Supplementary Table 2).

## Discussion

In this large, population-based cohort of patients with first-ever diagnosed PPD, we found the occurrence of treatment resistance to be common in patients with PPD. Moreover, our study pinpoints that multiple demographic and clinical profiles, including lower educational level, lower income, cigarette smoking, primiparity, cesarean section, preterm delivery (32–36 weeks) and pre-existing physical and psychiatric conditions, are independently associated with a higher risk of developing TRPPD.

In this study, the proportion of patients with PPD who fulfilled the predefined TRPPD was 6.0%. Even with stricter criteria applied in our study, the proportion of treatment resistance was higher than the proportion reported in a previous study conducted in the United States^22^. However, the numbers of peripartum depression (2.5%) and treatment resistance (5.0%) in the US study might have been underestimated because the study included only insured people and excluded women with prior psychosis, bipolar disorder or dementia^22^. By contrast, the proportion of treatment resistance in our study was lower than that reported among patients with non-perinatal depression in population-based studies in Sweden^12^ (13%) and Denmark^7^ (15%), and even lower than the proportions reported in clinical trials^10^ (30%). Several factors may explain the lower treatment resistance in PPD compared with non-perinatal depression. First, compared with patients with non-perinatal depression, some patients with PPD may hesitate to continue or initiate pharmacotherapy or choose psychotherapy over pharmacotherapy due to concerns of potential harmful effects on the infant through breastfeeding^6^; therefore, PPD patients may receive less pharmacotherapy during the study period. Indeed, our data showed that 13.5% of patients did not receive any pharmacological or ECT/rTMS treatment during the postpartum year. Thus, the low proportion might indicate that women with PPD are somewhat untreated or undertreated, which is supportive in previous reports^6,23^. Second, in 2010, a screening for PPD was introduced in Swedish national guidelines. Therefore, patients diagnosed from 2010 onward might have less severe symptoms; and timely treatments/interventions which, if successful, may contribute to the lower treatment resistance in our study. Our results indicate lower risks of TRPPD in patients diagnosed after 2010 than before 2010, which also supports this explanation. In addition, compared with previous studies in which patients were ascertained through inpatient/outpatient care diagnosis^7,12^, our study including primary care diagnosis may have captured more mild or moderate cases, leading to lower treatment resistance.

We found that several demographic factors, including lower educational attainment or household income, being non-cohabiting, being born outside Sweden and living in northern Sweden, were associated with higher risk of TRPPD. One potential interpretation is that socioeconomically disadvantaged patients may have less accessibility to healthcare resources and social support, resulting in deteriorating disease severity and treatment response. Similar findings have been observed for non-perinatal depression in previous studies^24^. For example, Gronemann et al.^25^ found that patients with depression becoming unemployed were at higher risk of treatment resistance. In addition, substantial evidence has indicated that low socioeconomic status was associated with elevated levels of depressive symptoms^26,27^, which also supports our findings.

Moreover, the results of higher risk of TRPPD among patients who were smoking is in line with findings in people with non-perinatal depression^24^. Possibly, smoking individuals have more severe depressive symptoms^28^, or smokers may receive bupropion for smoking cessation^29^; thus, they might receive more antidepressants while not being depressed. Besides, smoking is considered an indicator of unfavorable socioeconomic status in the Swedish female population^30^. These factors may explain the higher risk of TRPPD among smoking patients. It is also possible that smoking may interact with drug metabolism and affect treatment effect^31^, leading to more antidepressant treatments.

Although some studies indicate that multiparity may predict prolonged depressive symptoms^19^, most studies indicate that multiparity is associated with lower risk of PPD^32^. By contrast, in our data, PPD patients with two births (the most common type in Sweden) had a lower risk of TRPPD but not among patients with three or more births. However, in the sensitivity analysis excluding patients with prior psychiatric disorders, as well as in the mutually adjusted analysis, a higher risk of TRPPD was observed only among patients with three or more births. Thus, more research is needed to corroborate our findings on the link between parity and TRPPD. In addition, PPD patients who gave birth through cesarean section were more likely to develop PPD^33^. Cesarean section, particularly acute cesarean section, can lead to a negative delivery experience^20^, which may drive these patients to more severe symptoms of postpartum depression^21^. Preterm delivery at 32–36 weeks was associated with a higher risk of TRPPD. It is plausible that preterm birth entails higher risk of neonatal complications and stressful experiences such as admission to neonatal intensive care units^34^, which may contribute to worse prognosis of PPD. However, very preterm birth (22–31 weeks) was not associated with TRPPD, which argues against such explanation. In addition, low birth weight, another risk factor for neonatal complications, was not associated with TRPPD. We did not note a material difference in treatment response between patients with a live birth and those with a stillbirth, although the etiology of PPD may differ between these two groups, as depression following a stillbirth may be more closely related to grief over the loss.

Our finding on the higher risk of TRPPD among patients with previous physical conditions is also in line with studies on non-perinatal depression^15,35^. This could be attributed to the chronic inflammation, or altered hepatic or renal functions, or drug interactions with antidepressant due to these physical comorbidities^31^. Recent findings indicate non-responders to antidepressant treatment have higher inflammatory levels compared with responders^36^. Of note, we did not observe a higher risk of TRPPD among patients with gestational diabetes or pre-eclampsia, although mothers with such conditions generally have a higher rate of PPD^32,37^.

Pre-existing psychiatric conditions have been repeatedly and consistently associated with increased risk of treatment resistance in patients with non-perinatal depression^15,38^. In the study by Cepeda et al.^22^, women with perinatal depression who developed treatment resistance had higher rates of psychiatric comorbidities in the previous year than those who did not develop treatment resistance. However, the result is from descriptive comparisons without confounding adjustment^22^. In this study, we illustrated that pre-existing psychiatric conditions, both any psychiatric disorder and a range of specific psychiatric disorders, were associated with a higher risk of TRPPD. Patients with pre-existing psychiatric conditions might have received antidepressants to treat depression or conditions other than depression, and therefore could have become somewhat tolerant to antidepressants intended for their PPD^39^. By contrast, patients with prior premenstrual disorders were at a lower risk of TRPPD. This subgroup of patients are sensitive to normal changes of gonadal steroid levels^40^ and may represent a ‘hormone-sensitive’ phenotype of PPD^17^. Theoretically, in this subgroup, depressive symptoms are triggered by the normal drop of gonadal hormones after delivery, but symptoms may remit when hormone levels begin rising again. Future studies, however, are needed to elucidate the role of hormonal factors in treatment response of PPD. It is worth noting that when patients with a psychiatric history were excluded, we saw similar results for risk factors, including socioeconomic status, parity and prior physical comorbidities. This suggests these risk factors are independent of prior psychiatric conditions.

Of note, we found that most factors associated with TRPPD in our study overlap with the psychosocial risk factors for PPD. A probable explanation could be that PPD patients with these factors are more likely to develop more severe depression or require more intensive treatment due to pre-existing mental conditions, which increases their chances of developing treatment resistance or being classified as treatment resistant.

Several strengths of our study assure robustness of our findings. The nationwide population-based sample together with high-quality, prospectively collected healthcare register data enabled a representative sample of treatment resistance among women with PPD and a comprehensive assessment of various risk factors for TRPPD. Our study has weaknesses. First, in Sweden, depression is often managed in primary care^41^. While we used antidepressant prescriptions for identification, we might have missed mild PPD cases who were not prescribed pharmacotherapy as we only had primary care data from five counties. Yet this concern was addressed by the almost identical results when repeating the analysis using data from only these counties. Second, psychotherapy is the first-line treatment for mild to moderate depression in Sweden. However, due to lacking information on psychotherapy, the influence of psychotherapy on treatment response was not considered in our study. Future studies incorporating both psychotherapy and pharmacotherapy are highly warranted. Third, we may have some degree of misclassification of treatment resistance as our definition did not consider the dose of treatment. However, largely comparable results were obtained when utilizing two alternative definitions of treatment resistance. In addition, misclassification of treatment resistance may occur among patients with prior psychiatric disorders as these patients could restart or continue treatments for their prior illness during the postpartum period, leading to overestimation of TRPPD. The concern, however, was partially alleviated as we obtained similar results when excluding PPD patients who had any pre-existing psychiatric conditions. Last, our results might not be generalized to populations outside Sweden where different healthcare service systems apply.

In conclusion, based on a nationwide sample, our study shows that the prevalence of treatment resistance was common in patients with PPD. We found primiparous patients with lower socioeconomic status, cigarette smoking and pre-existing physical and psychiatric conditions are at a higher risk of developing treatment resistance. These findings may provide grounds for practical risk assessment at PPD diagnosis and highlight the need for personalized management strategies (for example, actively monitoring treatment response to aid earlier medication adjustments), particularly for patients identified as at risk for worse prognosis.

## Methods

### Data resource

This study was based on nationwide Swedish population and healthcare registers. Individuals were linked through the unique personal identification number that is assigned to every resident in Sweden. The Medical Birth Register (MBR)^42^ collects nationwide data on pregnancy, delivery and neonatal characteristics in Sweden from 1973. The National Patient Register^43^ includes nationwide information on inpatient specialized care since 1987 and on hospital-based outpatient specialized care since 2001 (coverage >80%). The Prescribed Drug Register^44^ includes all prescribed drugs dispensed at pharmacies since July 2005. We also included primary care data from five counties (Stockholm, Skåne, Uppsala, Värmland and Västra Götaland), which accounts for 58%–62% of women of reproductive age in Sweden during 2001–2021. The Longitudinal Integration Database for Health Insurance and Labor Market^45^ contains sociodemographic information, for example, income and education level, on a yearly basis for Swedish residents aged ≥16 years since 1990.

### Study design and study population

We conducted a nationwide, register-based cohort study leveraging a range of national health registers in Sweden. We first identified 1,042,423 women with 1,784,326 pregnancies from the MBR who gave birth during 2006–2021 in Sweden. After excluding 24,997 duplicate records of the same pregnancy due to multiple births and 31,090 pregnancies with erroneous records (that is, 31,083 possibly wrong identification, 7 died before childbirth), the study base consisted of 1,728,239 pregnancies from 1,022,698 women. We identified women with PPD from the study base and included only their first-ever PPD in cases of multiple PPD records. Further, we excluded patients with a record of antepartum depression for the pregnancy studied. Patients with psychosis, bipolar disorder or dementia between childbirth and PPD diagnosis were also excluded to ensure a sample of patients with definitive depression diagnosis.

The study was approved by the Swedish Ethics Review Authority (2018/1515-31 and 2021-02775). Informed consent from each participant was waived by Swedish law when using register-based data.

### Ascertainment of PPD

In line with previous studies^46^, we defined PPD as the first record of depression diagnosis in both specialist care (nationwide) and primary care (five counties available), or as a first filled prescription of antidepressants, during the first year after birth (identification codes are listed in Supplementary Table 3). While women with PPD can have a prior depression, it was the first identified PPD that was included in our analysis. Although in diagnostic definition^47^, PPD is defined as a depressive episode within weeks after childbirth, the timing is often extended to encompass the first year postpartum in research settings as the clinical diagnosis may be substantially delayed.

### Ascertainment of TRPPD

The outcome was treatment resistance occurring within 1 year following PPD diagnosis, that is, TRPPD. There is no consensus on definition of treatment-resistant depression. In addition, it is challenging to determine the reasons for medication changes or discontinuation in registry data as they could be due to side effects, lack of effectiveness or disease remission. Therefore, we adapted a treatment-resistant depression definition in previous studies^22^, which counts the number of antidepressants (that is, ≥3 different antidepressants) during 12 months. This definition has been proved to have better performance in discriminating individuals with and without evidence of treatment resistance than definitions that are based on adequacy of treatment dose and duration, and the definition achieves consistent performance across databases^48^. Besides, following Lundberg et al.^11^, we also considered use of add-on medication (lithium, risperidone, olanzapine, aripiprazole and quetiapine ( > 100 mg), which are recommended by guidelines for treatment-resistant depression^49^), or ECT or rTMS as treatment resistance. Briefly, we defined TRPPD as use of ≥3 different antidepressants, or add-on medication, or ECT or rTMS during the first year after the PPD diagnosis. Identification codes and data sources are listed in Supplementary Table 3.

### Potential risk factors for TRPPD

We sourced registers for demographics, lifestyle factors and pregnancy characteristics as potential risk factors. Maternal educational attainment and household income were obtained from the Longitudinal Integration Database for Health Insurance and Labor Market. Using the MBR, we retrieved information on maternal age, civil status, maternal birth country, maternal residency in Sweden, calendar year at delivery, and cigarette smoking, snus use and body mass index in early pregnancy. Pregnancy characteristics and pregnancy outcomes, including parity, multiple gestation, delivery mode, gestational length, birth weight, Apgar score at 5 min, stillbirth and gestational hypertensive or diabetic disorders, were also obtained from the MBR.

We considered pre-existing physical and psychiatric conditions (including any or specific categories of psychiatric disorders) before PPD diagnosis as potential risk factors and identified them from the National Patient Register. An adapted Charlson comorbidity index for register-based research in Sweden (we excluded diabetes as this category was listed separately in this study) was used as a proxy for physical comorbidity burden^50^. An index of zero indicates no comorbidity; the higher the index, the greater the comorbidity burden. Identification codes of psychiatric conditions are listed in Supplementary Table 3.

### Statistical analysis

#### Main analysis

We calculated the absolute risk of TRPPD using the number of TRPPD divided by total number of PPD patients during 1 year after PPD diagnosis. We used Poisson regression models to assess the potential risk factors associated with TRPPD by estimating RRs and 95% CIs, contrasting the absolute risk of TRPPD in the exposed group with that in the reference group. We started with univariable analysis (Model 1) followed by multivariable analysis (Model 2), which adjusted for maternal age, educational level, calendar year, residential region, maternal country of birth, parity and multiple gestation, whenever applicable. To address the concern of multiple testing as many risk factors were being tested, we collected the *P* values of all estimates and produced corresponding corrected *P* values using the false discovery rate (that is, Benjamini-Hochberg) method.

#### Sensitivity analyses

We performed several sensitivity analyses to test the robustness of our findings. First, a prior psychiatric condition may be an indication for treatments, including antidepressants, leading to being more likely to fulfill the criteria of TRPPD. Therefore, we limited the analyses to patients without a history of any psychiatric disorders. Second, some PPD cases were identified through antidepressants, which could have been prescribed for other psychiatric conditions (for example, anxiety disorders). We thus restricted the analysis to patients with a clinically confirmed diagnosis of PPD only. Third, we limited the analysis to counties with primary care data. Fourth, we used an alternative definition of TRPPD requiring at least one antidepressant before the add-on medication or ECT/rTMS: namely, use of ≥3 different antidepressants, or first antidepressant + add-on medication, or first antidepressant + ECT/rTMS during the year after the PPD diagnosis. Fifth, to consider potential misclassification of treatment resistance due to medication switches caused by side effects, we used an alternative TRPPD definition that accounted for duration of treatment. In this alternative definition, to count as an eligible drug trial during a treatment period (the time between two prescriptions for the same drug within 120 days^51^), another antidepressant or add-on medication had to be prescribed more than 28 days after the previous treatment started and last at least 28 days. Time-interval criteria were not applied to ECT or rTMS. Last, some of the risk factors studied might correlate with each other. We therefore included all factors in an additional model for mutual adjustment and assessed the independent factors associated with treatment resistance.

Data were processed and analyzed using SAS (version 9.4). All tests were two sided, and *P* < 0.05 was considered statistically significant.

### Reporting summary

Further information on research design is available in the Nature Portfolio Reporting Summary linked to this article.

## Supplementary information

Supplementary Information Supplementary Tables 1–3.

Reporting Summary

## Data Availability

Due to privacy protection measures, such as the General Data Protection Regulation (GDPR), the registers’ data are not publicly accessible. Researchers who are interested in replicating this study can apply for access to individual-level data through Statistics Sweden (https://www.scb.se/en/services/ordering-data-and-statistics/ordering-microdata/). Access to data on patient health can be applied for through Socialstyrelsen (https://www.socialstyrelsen.se/en/statistics-and-data/registers/).

## Code availability

Analysis coding is available via GitHub at https://github.com/YufChe/TRPPD.
