# Supplementary material for: Risk factors for treatment resistance among women with postpartum depression in a nationwide study
Source: Nat Ment Health. 2026 Feb 4;4(2):288–97. doi: 10.1038/s44220-026-00587-8 (PMC12890583; doi:10.1038/s44220-026-00587-8)
Supplement: Supplementary file 1 — Supplementary Tables 1–3. [file 44220_2026_587_MOESM1_ESM.pdf]

nature mental health

Supplementary information

https://doi.org/10.1038/s44220-026-00587-8

Risk factors for treatment resistance among women with postpartum depression in a nationwide study

In the format provided by the authors and unedited

Supplementary Table S1. Risk factors associated with TRPPD, restricted to patients without any pre-existing psychiatric disorders

	Non-TRPPD	TRPPD		RR (95% CI) [b]			
**Variables**	**(n=35057)**	**(n=1508)**	**AR (%)**	**Model 1**	***P*** [c]	**Model 2**	***P*** [c]
**Maternal age, y**							
<20	378 (1.1)	25 (1.7)	6.2	1.47 (0.98-2.19)	0.156	1.31 (0.86-2.00)	0.397
20-24	3780 (10.8)	225 (14.9)	5.6	1.33 (1.13-1.56)	0.002	1.22 (1.04-1.44)	0.075
25-29	10232 (29.2)	452 (30.0)	4.2	reference		reference	
30-34	12424 (35.4)	481 (31.9)	3.7	0.88 (0.77-1.00)	0.140	0.90 (0.79-1.03)	0.276
35-39	6674 (19.0)	264 (17.5)	3.8	0.90 (0.77-1.05)	0.348	0.88 (0.75-1.03)	0.276
≥40	1569 (4.5)	61 (4.0)	3.7	0.88 (0.68-1.16)	0.576	0.82 (0.62-1.08)	0.305
**Educational level, y**							
>12	17639 (50.3)	612 (40.6)	3.4	reference		reference	
9-12	13367 (38.1)	658 (43.6)	4.7	1.40 (1.25-1.56)	<0.001	1.26 (1.12-1.42)	0.003
<9	3219 (9.2)	180 (11.9)	5.3	1.58 (1.34-1.86)	<0.001	1.24 (1.03-1.49)	0.104
Unknown	832 (2.4)	58 (3.8)	6.5	NA		NA	
**Household income level**							
Top 20%	6770 (19.3)	228 (15.1)	3.3	reference		reference	
Middle	21950 (62.6)	966 (64.1)	4.2	1.29 (1.12-1.49)	0.002	1.17 (1.01-1.35)	0.139
Lowest 20%	6086 (17.4)	293 (19.4)	4.6	1.41 (1.19-1.68)	0.001	1.18 (0.98-1.41)	0.220
Unknown	251 (0.7)	21 (1.4)	7.7	NA		NA	
**Civil status**							
Cohabiting	32888 (93.8)	1399 (92.8)	4.1	reference		reference	
Non-cohabiting	2169 (6.2)	109 (7.2)	4.8	1.17 (0.97-1.43)	0.237	1.04 (0.85-1.27)	0.869
**Calendar year at delivery**							
2006-2010	9781 (27.9)	478 (31.7)	4.7	reference		reference	
2010-2015	10870 (31.0)	508 (33.7)	4.5	0.96 (0.85-1.09)	0.662	0.98 (0.86-1.11)	0.869
2015-2021	14406 (41.1)	522 (34.6)	3.5	0.75 (0.66-0.85)	<0.001	0.78 (0.69-0.89)	0.003
**Maternal country of birth**							
Sweden	28268 (80.6)	1144 (75.9)	3.9	reference		reference	
Europe	2410 (6.9)	129 (8.6)	5.1	1.31 (1.09-1.57)	0.014	1.32 (1.10-1.60)	0.024
Other	4379 (12.5)	235 (15.6)	5.1	1.31 (1.14-1.51)	0.001	1.29 (1.11-1.50)	0.015
**Maternal residency in Sweden**							
Southern	7509 (21.4)	332 (22.0)	4.2	reference		reference	
Middle	21682 (61.8)	875 (58.0)	3.9	0.92 (0.81-1.04)	0.348	0.95 (0.83-1.07)	0.598
Northern	5866 (16.7)	301 (20.0)	4.9	1.15 (0.99-1.35)	0.177	1.15 (0.99-1.35)	0.214
**BMI in early pregnancy, kg/m²**							
<18.5	754 (2.2)	45 (3.0)	5.6	1.43 (1.06-1.93)	0.055	1.32 (0.98-1.79)	0.214
18.5-<25	17953 (51.2)	736 (48.8)	3.9	reference		reference	
25-<30	8843 (25.2)	375 (24.9)	4.1	1.03 (0.91-1.17)	0.742	1.00 (0.88-1.13)	0.999
30-<40	4702 (13.4)	235 (15.6)	4.8	1.21 (1.04-1.40)	0.036	1.13 (0.97-1.31)	0.276
≥40	488 (1.4)	16 (1.1)	3.2	0.81 (0.49-1.32)	0.597	0.73 (0.45-1.21)	0.400
Unknown	2317 (6.6)	101 (6.7)	4.2	NA		NA	
**Smoking in early pregnancy**							
No smoking	30943 (88.3)	1267 (84.0)	3.9	reference		reference	
1-9 cigarettes/day	1743 (5.0)	118 (7.8)	6.3	1.61 (1.33-1.95)	<0.001	1.35 (1.11-1.64)	0.023
≥10 cigarettes/day	515 (1.5)	37 (2.5)	6.7	1.70 (1.23-2.36)	0.005	1.41 (1.01-1.97)	0.149
Unknown	1856 (5.3)	86 (5.7)	4.4	NA		NA	
**Snuff use in early pregnancy**							
No	33158 (94.6)	1412 (93.6)	4.1	reference		reference	
Yes	640 (1.8)	34 (2.3)	5.0	0.81 (0.58-1.14)	0.414	0.89 (0.63-1.25)	0.710
Unknown	1259 (3.6)	62 (4.1)	4.7	NA		NA	
**Parity**							
1	15072 (43.0)	617 (40.9)	3.9	reference		reference	
2	13589 (38.8)	558 (37.0)	3.9	1.00 (0.89-1.12)	0.999	1.04 (0.92-1.17)	0.719
≥3	6396 (18.2)	333 (22.1)	4.9	1.26 (1.10-1.44)	0.003	1.27 (1.09-1.47)	0.020

**Multiple gestation**							
No	34396 (98.1)	1479 (98.1)	4.1	reference		reference	
Yes	661 (1.9)	29 (1.9)	4.2	1.02 (0.71-1.47)	0.977	1.05 (0.73-1.52)	0.915
**Delivery mode**							
Non-assisted vaginal	25271 (72.1)	1075 (71.3)	4.1	reference		reference	
Assisted vaginal	2144 (6.1)	84 (5.6)	3.8	0.92 (0.74-1.15)	0.656	0.99 (0.79-1.24)	0.999
Caesarean-section	7642 (21.8)	349 (23.1)	4.4	1.07 (0.95-1.21)	0.465	1.12 (0.99-1.27)	0.214
**Gestational length, weeks**							
22-31	528 (1.5)	16 (1.1)	2.9	0.72 (0.44-1.18)	0.361	0.70 (0.43-1.15)	0.305
32-36	1764 (5.0)	85 (5.6)	4.6	1.12 (0.90-1.40)	0.489	1.11 (0.89-1.39)	0.551
37-41	30785 (87.8)	1315 (87.2)	4.1	reference		reference	
42-46	1975 (5.6)	92 (6.1)	4.5	1.09 (0.88-1.34)	0.640	1.11 (0.90-1.38)	0.526
Unknown	5 (<0.1)	<5 (<0.1)	<0.1	NA		NA	
**Birth weight, g**							
<1500	465 (1.3)	12 (0.8)	2.5	0.61 (0.34-1.07)	0.196	0.60 (0.34-1.06)	0.218
1500-<2500	1234 (3.5)	59 (3.9)	4.6	1.10 (0.85-1.43)	0.640	1.08 (0.82-1.41)	0.776
≥2500	33296 (95)	1437 (95.3)	4.1	reference		reference	
Unknown	62 (0.2)	<5 (<0.1)	<0.1	NA		NA	
**Apgar score at 5 min**							
≥7	33894 (96.7)	1462 (96.9)	4.1	reference		reference	
<7	893 (2.5)	37 (2.5)	4.0	0.96 (0.69-1.33)	0.907	0.94 (0.68-1.31)	0.869
Unknown	270 (0.8)	9 (0.6)	3.2	NA		NA	
**Stillbirth**							
No	34746 (99.1)	1499 (99.4)	4.1	reference		reference	
Yes	311 (0.9)	9 (0.6)	2.8	0.68 (0.35-1.31)	0.444	0.62 (0.32-1.19)	0.305
**Hypertension**							
No	33510 (95.6)	1437 (95.3)	4.1	reference		reference	
Essential hypotension	267 (0.8)	13 (0.9)	4.6	1.13 (0.65-1.95)	0.789	1.15 (0.66-1.98)	0.802
Preeclampsia	1280 (3.7)	58 (3.8)	4.3	1.05 (0.81-1.37)	0.806	1.10 (0.84-1.43)	0.710
**Diabetes**							
No	34104 (97.3)	1464 (97.1)	4.1	reference		reference	
Gestational diabetes	685 (2.0)	33 (2.2)	4.6	1.12 (0.79-1.58)	0.681	1.12 (0.79-1.58)	0.720
Pregestational diabetes	268 (0.8)	11 (0.7)	3.9	0.96 (0.53-1.73)	0.964	0.94 (0.52-1.71)	0.964
**Charlson comorbidity**							
0	31667 (90.3)	1322 (87.7)	4.0	reference		reference	
1	2769 (7.9)	147 (9.7)	5.0	1.26 (1.06-1.49)	0.028	1.28 (1.08-1.52)	0.028
≥2	621 (1.8)	39 (2.6)	5.9	1.47 (1.07-2.03)	0.050	1.52 (1.10-2.09)	0.056
**Premenstrual disorder**							
No	33214 (94.7)	1466 (97.2)	4.2	reference		reference	
Yes	1843 (5.3)	42 (2.8)	2.2	0.53 (0.39-0.72)	<0.001	0.61 (0.45-0.84)	0.020

TRPPD, treatment-resistant postpartum depression; AR, absolute risk; RR, risk ratio; CI, confidence interval; BMI, body mass index; NA, not applicable.
[a] AR% = (number of TRPPD / total number of patients with PPD in this category) * 100%.
[b] Model 1: estimates from Poisson regression, univariate analysis without adjustment.
Model 2: estimates from Poisson regression, adjusted for age, educational level, calendar year, residential region, maternal country of birth, parity, and multiple gestation, if applicable.
[c] *P* values were corrected for multiple testing using Benjamini and Hochberg method.

Supplementary Table S2. Risk factors associated with TRPPD, sensitivity analyses

	**Clinically diagnosed PPD patients** [a]	**PPD patients from regions with primary care data** [b]	**Alternative definition of TRPPD** [c]	**Alternative definition of TRPPD** [d]	**Mutually adjustment model** [e]
	Non-TRPPD / TRPPD (AR% [f])	Non-TRPPD / TRPPD (AR% [f])	Non-TRPPD / TRPPD (AR% [f])	Non-TRPPD / TRPPD (AR% [f])	Non-TRPPD / TRPPD (AR% [f])
	21873 / 1799 (7.60%)	34932 / 1975 (5.35%)	55353 / 3265 (5.57%)	54186 / 4432 (7.56%)	55096 / 3522 (6.01%)
**Maternal age, y**					
<20	0.95 (0.68-1.33)	1.16 (0.82-1.63)	1.10 (0.86-1.40)	0.94 (0.74-1.19)	1.16 (0.91-1.47)
20-24	1.11 (0.96-1.29)	1.09 (0.94-1.27)	1.09 (0.98-1.22)	0.97 (0.88-1.07)	1.11 (1.00-1.24)
25-29	reference	reference	reference	reference	reference
30-34	1.02 (0.90-1.16)	0.94 (0.84-1.06)	0.96 (0.88-1.06)	1.03 (0.96-1.12)	0.94 (0.86-1.03)
35-39	1.02 (0.87-1.18)	1.04 (0.91-1.19)	1.01 (0.91-1.13)	1.02 (0.93-1.12)	0.96 (0.86-1.06)
≥40	1.12 (0.88-1.43)	1.19 (0.96-1.46)	1.00 (0.83-1.20)	1.14 (0.98-1.33)	0.92 (0.77-1.10)
**Educational level, y**					
>12	reference	reference	reference	reference	reference
9-12	1.27 (1.13-1.42)	1.24 (1.11-1.38)	1.25 (1.15-1.35)	1.10 (1.03-1.18)	1.09 (1.00-1.18)
<9	1.56 (1.34-1.82)	1.63 (1.41-1.88)	1.61 (1.44-1.80)	1.19 (1.07-1.31)	1.15 (1.02-1.29)
**Household income level**					
Top 20%	reference	reference	reference	reference	reference
Middle	1.23 (1.07-1.41)	1.14 (1.01-1.29)	1.20 (1.08-1.33)	1.14 (1.05-1.24)	1.14 (1.03-1.26)
Lowest 20%	1.32 (1.13-1.56)	1.36 (1.17-1.58)	1.35 (1.19-1.52)	1.18 (1.06-1.31)	1.19 (1.06-1.34)
**Civil status**					
Cohabiting	reference	reference	reference	reference	reference
Non-cohabiting	1.19 (1.02-1.39)	1.28 (1.11-1.48)	1.24 (1.10-1.38)	1.12 (1.01-1.25)	1.04 (0.93-1.16)
**Calendar year at delivery**					
2006-2010	reference	reference	reference	reference	reference
2010-2015	0.88 (0.78-0.98)	0.95 (0.85-1.06)	0.98 (0.89-1.07)	0.85 (0.79-0.91)	0.94 (0.87-1.03)
2015-2021	0.76 (0.68-0.85)	0.77 (0.69-0.86)	0.85 (0.78-0.92)	0.74 (0.68-0.79)	0.81 (0.74-0.88)
**Maternal country of birth**					
Sweden	reference	reference	reference	reference	reference
Europe	1.24 (1.04-1.47)	1.14 (0.96-1.35)	1.18 (1.03-1.36)	0.98 (0.88-1.06)	1.31 (1.15-1.49)
Other	1.09 (0.95-1.25)	1.20 (1.05-1.36)	1.12 (1.00-1.24)	1.14 (1.01-1.32)	1.21 (1.08-1.34)
**Maternal residency in Sweden**					
Southern	reference	reference	reference	reference	reference
Middle	0.95 (0.85-1.07)	1.10 (0.98-1.22)	1.00 (0.92-1.09)	0.95 (0.88-1.02)	1.02 (0.94-1.11)
Northern	2.15 (1.83-2.52)	1.00 (0.79-1.26)	1.18 (1.05-1.31)	1.14 (1.04-1.25)	1.20 (1.08-1.33)
**BMI in early pregnancy, kg/m^2**					
<18.5	1.13 (0.85-1.50)	1.23 (0.94-1.59)	1.13 (0.91-1.41)	1.06 (0.87-1.29)	1.16 (0.95-1.43)
18.5-<25	reference	reference	reference	reference	reference
25-<30	0.99 (0.88-1.11)	1.02 (0.91-1.14)	1.02 (0.93-1.11)	1.04 (0.97-1.12)	0.98 (0.90-1.06)
30-<40	1.05 (0.91-1.21)	1.04 (0.91-1.20)	1.09 (0.99-1.21)	1.14 (1.05-1.25)	1.00 (0.91-1.11)
≥40	0.86 (0.55-1.32)	1.08 (0.73-1.58)	0.99 (0.75-1.30)	0.80 (0.61-1.05)	0.88 (0.67-1.14)
**Smoking in early pregnancy**					
No smoking	reference	reference	reference	reference	reference
1-9 cigarettes/day	1.31 (1.10-1.55)	1.48 (1.27-1.73)	1.44 (1.28-1.62)	1.33 (1.19-1.48)	1.24 (1.11-1.39)
≥10 cigarettes/day	1.55 (1.19-2.03)	1.63 (1.28-2.08)	1.45 (1.20-1.76)	1.27 (1.06-1.52)	1.23 (1.02-1.48)
**Snuff use in early pregnancy**					
No	reference	reference	reference	reference	reference
Yes	0.79 (0.58-1.09)	0.93 (0.65-1.33)	0.92 (0.74-1.14)	0.99 (0.81-1.20)	0.98 (0.80-1.21)
**Parity**					
1	reference	reference	reference	reference	reference
2	0.89 (0.80-0.99)	0.88 (0.79-0.97)	0.91 (0.84-0.99)	0.96 (0.89-1.02)	1.02 (0.94-1.10)

≥3	0.99 (0.86-1.13)	0.98 (0.86-1.12)	1.03 (0.93-1.14)	0.98 (0.90-1.07)	1.16 (1.05-1.29)
**Multiple gestation**					
No	reference	reference	reference	reference	reference
Yes	1.01 (0.72-1.41)	0.97 (0.70-1.35)	1.03 (0.80-1.32)	1.00 (0.81-1.24)	0.95 (0.74-1.23)
**Delivery mode**					
Non-assisted vaginal	reference	reference	reference	reference	reference
Assisted vaginal	0.95 (0.77-1.16)	0.99 (0.82-1.20)	1.01 (0.87-1.17)	1.02 (0.90-1.15)	1.00 (0.87-1.16)
Caesarean-section	1.14 (1.02-1.27)	1.20 (1.08-1.33)	1.15 (1.06-1.25)	1.11 (1.03-1.19)	1.10 (1.02-1.20)
**Gestational length, weeks**					
22-31	0.92 (0.64-1.33)	0.90 (0.63-1.29)	0.92 (0.69-1.22)	0.85 (0.67-1.25)	1.38 (0.87-2.19)
32-36	1.24 (1.03-1.49)	1.22 (1.01-1.46)	1.23 (1.07-1.41)	1.17 (1.02-1.34)	1.21 (1.04-1.42)
37-41	reference	reference	reference	reference	reference
42-46	1.05 (0.87-1.27)	0.94 (0.77-1.14)	1.00 (0.85-1.16)	1.07 (0.94-1.21)	1.01 (0.87-1.17)
**Birth weight, g**					
<1500	0.71 (0.46-1.09)	0.79 (0.52-1.20)	0.81 (0.59-1.12)	0.82 (0.62-1.08)	0.54 (0.32-0.91)
1500-<2500	1.09 (0.87-1.37)	1.12 (0.90-1.39)	1.08 (0.91-1.28)	1.00 (0.86-1.17)	0.89 (0.73-1.09)
≥2500	reference	reference	reference	reference	reference
**Apgar score at 5 min**					
≥7	reference	reference	reference	reference	reference
<7	1.13 (0.88-1.45)	1.02 (0.78-1.32)	1.03 (0.84-1.26)	0.93 (0.77-1.12)	1.04 (0.83-1.30)
**Stillbirth**					
No	reference	reference	reference	reference	reference
Yes	0.95 (0.64-1.43)	0.83 (0.53-1.31)	0.84 (0.59-1.22)	0.86 (0.62-1.18)	0.94 (0.63-1.41)
**Hypertension**					
No	reference	reference	reference	reference	reference
Essential hypotension	1.37 (0.84-2.24)	1.08 (0.67-1.75)	1.13 (0.79-1.64)	1.08 (0.79-1.49)	1.13 (0.79-1.62)
Preeclampsia	1.16 (0.92-1.47)	1.15 (0.92-1.43)	1.10 (0.93-1.31)	1.17 (1.02-1.36)	1.13 (0.95-1.34)
**Diabetes**					
No	reference	reference	reference	reference	reference
Gestational diabetes	1.05 (0.75-1.47)	0.95 (0.69-1.31)	1.12 (0.88-1.40)	1.09 (0.88-1.33)	1.00 (0.80-1.26)
Pregestational diabetes	0.66 (0.34-1.26)	0.38 (0.17-0.84)	0.64 (0.41-1.01)	0.84 (0.60-1.18)	0.59 (0.39-0.92)
**Charlson comorbidity index**					
0	reference	reference	reference	reference	reference
1	1.25 (1.07-1.45)	1.24 (1.08-1.43)	1.27 (1.15-1.42)	1.14 (1.04-1.26)	1.15 (1.04-1.28)
≥2	1.64 (1.25-2.15)	1.32 (1.00-1.73)	1.46 (1.20-1.78)	1.20 (1.00-1.45)	1.24 (1.02-1.51)
**Psychiatric disorder, any**					
No	reference	reference	reference	reference	reference
Yes	1.90 (1.73-2.09)	2.33 (2.13-2.56)	2.15 (2.00-2.31)	1.53 (1.44-1.63)	1.67 (1.52-1.83)
**Psychotic disorders**					
No	reference	reference	reference	reference	reference
Yes	6.83 (5.74-8.13)	9.53 (8.23-11.03	7.71 (6.82-8.72)	4.98 (4.38-5.67)	4.76 (4.20-5.39)
**Sleep disorders**					
No	reference	reference	reference	reference	reference
Yes	1.80 (1.33-2.44)	2.18 (1.64-2.89)	2.01 (1.63-2.48)	1.62 (1.32-1.98)	1.14 (0.93-1.40)
**Personality disorder**					
No	reference	reference	reference	reference	reference
Yes	1.47 (1.18-1.83)	2.05 (1.70-2.47)	2.03 (1.76-2.33)	1.53 (1.33-1.75)	0.96 (0.82-1.11)
**Developmental and/or neuropsychiatric disorders**					
No	reference	reference	reference	reference	reference
Yes	1.46 (1.22-1.75)	1.77 (1.49-2.09)	1.71 (1.52-1.94)	1.30 (1.15-1.47)	1.00 (0.88-1.13)
**Depressive disorder**					
No	reference	reference	reference	reference	reference
Yes	1.38 (1.23-1.55)	1.78 (1.59-1.98)	1.68 (1.54-1.83)	1.40 (1.29-1.51)	0.95 (0.86-1.04)
**Anxiety**					
No	reference	reference	reference	reference	reference
Yes	1.67 (1.50-1.87)	1.88 (1.70-2.08)	1.74 (1.61-1.89)	1.43 (1.33-1.53)	1.04 (0.95-1.14)
**Substance abuse**					

No	reference	reference	reference	reference	reference
Yes	1.32 (1.12-1.55)	1.82 (1.59-2.09)	1.65 (1.48-1.83)	1.35 (1.22-1.49)	0.98 (0.88-1.09)
**Stress-related disorders**					
No	reference	reference	reference	reference	reference
Yes	1.58 (1.39-1.79)	1.79 (1.59-2.01)	1.69 (1.54-1.85)	1.31 (1.20-1.43)	1.06 (0.96-1.16)
**Premenstrual disorder**					
No	reference	reference	reference	reference	reference
Yes	1.04 (0.78-1.37)	0.80 (0.64-1.01)	0.73 (0.60-0.89)	1.05 (0.92-1.21)	0.72 (0.59-0.87)

TRPPD, treatment-resistant postpartum depression; AR, absolute risk; ECT/rTMS, electroconvulsive therapy or repetitive transcranial magnetic stimulation.

All estimates from Poisson regression, adjusted for age, educational level, calendar year, residential region, maternal country of birth, parity, and multiple gestation, if applicable.

[a] Restricted to PPD patients with a clinically confirmed depression diagnosis from the Patient register or primary care registers.

[b] Restricted to PPD patients in the counties having primary care data.

[c] Alternative definition of TRPPD: use of either ≥3 different antidepressants, or first antidepressant + add-on medication, or first antidepressant + ECT/rTMS during one year after the PPD diagnosis.

[d] Alternative definition of TRPPD: use of either ≥3 antidepressants, or first antidepressant + add-on medication, or first antidepressant + ECT/rTMS during one year after the PPD diagnosis. To qualify as an eligible drug trial (includes new treatment with the same ATC code) in a treatment period (defined as the time interval between any two consecutive prescriptions for the same drug within 120 days), another antidepressant or add-on medication had to be prescribed more than 28 days after the previous treatment initiation and have a duration of 28 days or longer for dosing.

[e] Estimates from Poisson regression, mutually adjusted for all variables listed in this table.

[f] AR% = (number of TRPPD / total number of patients with PPD) * 100%.

Supplementary Table S3. Ascertainment of postpartum depression, treatments, and psychiatric conditions

	**Identification codes**	**Data source**
**Postpartum depression (PPD):** defined as a first record of depression diagnosis or a first filled prescription of antidepressants from delivery to one year postpartum	ICD-9: 300E, 311; ICD-10: F32, F33, F34, F38, F39, F530	National Patient Register
	ICD-10: F32, F33, F34, F38, F39, F530, F53-	Primary care register
	ATC: N06A	Prescribed Drug Register
**Treatments**		
Antidepressants	ATC: N06A	Prescribed Drug Register
Add-on medications	ATC: N05AH03, N05AH04 (>100 mg), N05AX08, N05AX12, N05AN01	Prescribed Drug Register
Electroconvulsive therapy (ECT)	Clinical procedure codes: DA006, DA024, DA025	National Patient Register
Repetitive transcranial magnetic stimulation (rTMS)	Clinical procedure codes: DU050	National Patient Register
**Psychiatric conditions**		
Any	ICD-9: 290-315; ICD-10: F10-F99	National Patient Register
Developmental and/or neuropsychiatric disorders	ICD-9: 299, 307, 314, 315, 317-319; ICD-10: F70-F79, F80-F89, F90	National Patient Register
Depressive disorder	ICD codes: the same as PPD above	National Patient Register
Psychotic disorders	ICD-9: 295, 297, 298; ICD-10: F20-25, F28-29, F531	National Patient Register
Bipolar disorders	ICD-9: 296A, 296C, 296D, 296E, 296W, 296X<br>ICD-10: F30, F31	National Patient Register
Dementia	ICD-9: 290, 294; ICD-10: F00-F03	National Patient Register
Anxiety	ICD-9: 300A, 300C<br>ICD-10: F400-F402, F408, F409, F410-F413, F418, F419	National Patient Register
Stress-related disorders	ICD-9: 308, 309; ICD-10: F43	National Patient Register
Substance abuse	ICD-9: 291, 292, 303, 304; ICD-10: F10-F16, F18-F19	National Patient Register
Sleep disorders	ICD-9: 780F, 307E; ICD-10: G47, F51	National Patient Register
Personality disorders	ICD-9: 301; ICD-10: F60	National Patient Register
Premenstrual disorder	ICD-9: 625E; ICD-10: N943	National Patient Register

ICD codes, International Classification of Diseases codes; ATC codes, Anatomical Therapeutic Chemical codes.
