# Supplementary material for: Risk factors for treatment resistance among women with postpartum depression in a nationwide study
Source: Nat Ment Health. 2026 Feb 4;4(2):288–97. doi: 10.1038/s44220-026-00587-8 (PMC12890583; doi:10.1038/s44220-026-00587-8)
Supplement: Supplementary file 2 — Reporting Summary [file 44220_2026_587_MOESM2_ESM.pdf]

nature portfolio

Corresponding author(s): Yufeng Chen, Donghao Lu

Last updated by author(s): Dec 12, 2025

Reporting Summary

Nature Portfolio wishes to improve the reproducibility of the work that we publish. This form provides structure for consistency and transparency in reporting. For further information on Nature Portfolio policies, see our Editorial Policies and the Editorial Policy Checklist.

Statistics

For all statistical analyses, confirm that the following items are present in the figure legend, table legend, main text, or Methods section.

n/a	Confirmed	
☐	☒	The exact sample size (n) for each experimental group/condition, given as a discrete number and unit of measurement
☐	☒	A statement on whether measurements were taken from distinct samples or whether the same sample was measured repeatedly
☐	☒	The statistical test(s) used AND whether they are one- or two-sided<br>*Only common tests should be described solely by name; describe more complex techniques in the Methods section.*
☐	☒	A description of all covariates tested
☐	☒	A description of any assumptions or corrections, such as tests of normality and adjustment for multiple comparisons
☐	☒	A full description of the statistical parameters including central tendency (e.g. means) or other basic estimates (e.g. regression coefficient) AND variation (e.g. standard deviation) or associated estimates of uncertainty (e.g. confidence intervals)
☐	☒	For null hypothesis testing, the test statistic (e.g. F, t, r) with confidence intervals, effect sizes, degrees of freedom and P value noted<br>*Give P values as exact values whenever suitable.*
☒	☐	For Bayesian analysis, information on the choice of priors and Markov chain Monte Carlo settings
☒	☐	For hierarchical and complex designs, identification of the appropriate level for tests and full reporting of outcomes
☒	☐	Estimates of effect sizes (e.g. Cohen's d, Pearson's r), indicating how they were calculated

Our web collection on statistics for biologists contains articles on many of the points above.

Software and code

Policy information about availability of computer code

Data collection	No software was used for data collection.
Data analysis	All data analyses were conducted using SAS (version 9.4, SAS Institute). Analysis coding is available via GitHub at https://github.com/YufChe/TRPPD.

For manuscripts utilizing custom algorithms or software that are central to the research but not yet described in published literature, software must be made available to editors and reviewers. We strongly encourage code deposition in a community repository (e.g. GitHub). See the Nature Portfolio guidelines for submitting code & software for further information.

Data

Policy information about availability of data

All manuscripts must include a data availability statement. This statement should provide the following information, where applicable:

- Accession codes, unique identifiers, or web links for publicly available datasets
- A description of any restrictions on data availability
- For clinical datasets or third party data, please ensure that the statement adheres to our policy

Due to privacy protection measures, such as the General Data Protection Regulation (GDPR), the registers data is not publicly accessible. Researchers who are interested in replicating this study can apply for access to individual-level data through Statistics Sweden (https://www.scb.se/ en/services/ordering-data-and-

statistics/ordering-microdata/). Access to data on patient health can be applied for through Socialstyrelsen (https://www.socialstyrelsen.se/en/statistics-and-data/registers/).

Research involving human participants, their data, or biological material

Policy information about studies with human participants or human data. See also policy information about sex, gender (identity/presentation), and sexual orientation and race, ethnicity and racism.

Reporting on sex and gender	The study participants only included female patients with a diagnosis of postpartum depression. We used the term women in alignment with the literature, although individuals may identify differently.
Reporting on race, ethnicity, or other socially relevant groupings	The socially relevant variables in this study included educational attainment and household income, civil status, maternal birth country, and maternal residency in Sweden. We retrieved information on these variables from Swedish population and healthcare registers.<br>In the multivariable analysis, we included maternal age, educational level, calendar year, residential region, maternal country of birth, parity, and multiple gestation to control for confounding.
Population characteristics	Among the 58,618 postpartum depression patients included in the final analysis (mean age 30.8, SD 5.3 years), 50,679 (86.5%) had received treatments (either antidepressants, or add-on medications, or ECT/rTMS), and 3,522 (6.0%) fulfilled the criteria of treatment resistance within one year after postpartum depression diagnosis.
Recruitment	This study was based on nationwide Swedish population and healthcare registers. Individuals were linked through the unique personal identification number which is assigned to every resident in Sweden. The study participants were identified from the Medical Birth Register who gave birth during 2006-2021 in Sweden.
Ethics oversight	The study was approved by the Swedish Ethics Review Authority (2018/1515-31 and 2021-02775). Informed consent to each participant was waived by Swedish law when using register-based data.

Note that full information on the approval of the study protocol must also be provided in the manuscript.

Field-specific reporting

Please select the one below that is the best fit for your research. If you are not sure, read the appropriate sections before making your selection.

☐ Life sciences ☒ Behavioural & social sciences ☐ Ecological, evolutionary & environmental sciences

For a reference copy of the document with all sections, see nature.com/documents/nr-reporting-summary-flat.pdf

Behavioural & social sciences study design

All studies must disclose on these points even when the disclosure is negative.

Study description	We conducted a nationwide, register-based cohort study with quantitative data in Sweden.
Research sample	We first identified pregnant women from the Medical Birth Register who gave birth and diagnosed with a postpartum depression during 2006-2021 in Sweden.
Sampling strategy	This study was based on nationwide Swedish population and healthcare registers. The study participants were identified from the Medical Birth Register who gave birth during 2006-2021 in Sweden.
Data collection	This study was based on nationwide Swedish population and healthcare registers. Data used for this study was retrieved from a range of national registers.
Timing	The study population included women who gave birth during 2006-2021 in Sweden.
Data exclusions	We first identified 1,042,423 women with 1,784,326 pregnancies from the Medical Birth Register who gave birth during 2006-2021 in Sweden. After excluding 24,997 duplicate records of the same pregnancy due to multiple births, and 31,090 pregnancies with erroneous records, the study base consisted of 1,728,239 pregnancies from 1,022,698 women. In total, we identified 64,150 patients with a diagnosis of first-ever postpartum depression. After excluding 5,441 patients with a record of antepartum depression and 91 with psychosis, bipolar disorder, or dementia between childbirth and postpartum depression diagnosis, 58,618 patients remained for the final analysis.
Non-participation	This is register-based study. No participants declined participation.
Randomization	This is an observational study and no randomization was done. In the multivariable analysis, we included maternal age, educational level, calendar year, residential region, maternal country of birth, parity, and multiple gestation to control for confounding.

Reporting for specific materials, systems and methods

We require information from authors about some types of materials, experimental systems and methods used in many studies. Here, indicate whether each material, system or method listed is relevant to your study. If you are not sure if a list item applies to your research, read the appropriate section before selecting a response.

Materials & experimental systems

n/a	Involved in the study
☒	☐ Antibodies
☒	☐ Eukaryotic cell lines
☒	☐ Palaeontology and archaeology
☒	☐ Animals and other organisms
☒	☐ Clinical data
☒	☐ Dual use research of concern
☒	☐ Plants

Methods

n/a	Involved in the study
☒	☐ ChIP-seq
☒	☐ Flow cytometry
☒	☐ MRI-based neuroimaging

Plants

Seed stocks	No plant materials were used in this study.
Novel plant genotypes	No plant materials were used in this study.
Authentication	No plant materials were used in this study.
